# Role of Transvaginal Sonography in the Diagnosis of Female Infertility: A Comprehensive Review

**DOI:** 10.7759/cureus.50048

**Published:** 2023-12-06

**Authors:** Nirja Thaker, Rajasbala Dhande, Pratapsingh Parihar

**Affiliations:** 1 Radiology, Jawaharlal Nehru Medical College, Datta Meghe Institute of Higher Education and Research, Wardha, IND; 2 Radiodiagnosis, Jawaharlal Nehru Medical College, Datta Meghe Institute of Higher Education and Research, Wardha, IND

**Keywords:** early intervention, structural abnormalities, ovulatory disorders, diagnosis, transvaginal sonography, female infertility

## Abstract

Female infertility, a complex and emotionally challenging condition, impacts millions of women worldwide. Timely and accurate diagnosis is crucial for tailoring effective solutions to overcome fertility challenges. Transvaginal sonography, a real-time and non-invasive imaging modality, is pivotal in this diagnostic process. This review focuses on the structural abnormalities of the female reproductive system related to female infertility, particularly highlighting the capabilities of transvaginal sonography in assessing ovulatory disorders, structural anomalies, endometrial conditions, ovarian reserve, and other contributing factors. It is important to note that while transvaginal sonography excels in detecting structural abnormalities, it may not effectively identify lifestyle and hormonal changes. This limitation underscores the necessity for a comprehensive diagnostic approach that includes additional modalities to address the multifaceted nature of female infertility. Despite acknowledging the inherent limitations and operator dependence of transvaginal sonography, we emphasize its significance in guiding clinicians toward well-informed decisions and personalized treatment plans. Looking forward, we anticipate the continual evolution of sonographic technology, offering enhanced diagnostic capabilities. The commitment to improving fertility outcomes for individuals and couples navigating the intricate path toward parenthood remains paramount. In conclusion, a holistic diagnostic approach incorporating various modalities is essential for a thorough understanding and effective management of female infertility.

## Introduction and background

Infertility, in the context of female reproductive health, refers to the inability of a woman to conceive and carry a pregnancy to term despite engaging in regular unprotected sexual intercourse for an extended period, typically one year. This condition can be emotionally and psychologically distressing for individuals and couples wanting children. Female infertility can arise from various factors, including ovulatory disorders, structural abnormalities of the reproductive organs, endometrial issues, hormonal imbalances, and age-related factors. Understanding the definition of female infertility is essential to address its significant impact on individuals and society [1-3].

The prevalence of female infertility is a global concern. Around 17.5% of the adult population - roughly 1 in 6 worldwide - experience infertility, showing the urgent need to increase access to affordable, high-quality fertility care for those in need. It affects millions of women worldwide, becoming a widespread reproductive health issue. The prevalence varies across regions and populations, influenced by age, lifestyle, and underlying medical conditions. In this comprehensive review, we will explore the various factors contributing to female infertility and the role of transvaginal sonography in its diagnosis [4].

Early and accurate diagnosis of female infertility is paramount for several reasons. First, it allows for timely interventions and fertility treatments, increasing the chances of successful conception. Second, it can help identify underlying medical conditions or reproductive health issues requiring medical or surgical management. Third, early diagnosis can alleviate emotional distress for individuals and couples by clarifying the reasons for fertility struggles. Early diagnosis can save time and reduce the financial burden of prolonged infertility investigations [5]. Timely diagnosis is particularly crucial given the potential age-related decline in female fertility. As women age, the quality and quantity of their eggs diminish, making it increasingly challenging to conceive. Therefore, a prompt and accurate diagnosis can enable healthcare providers to tailor treatment options to individual needs, potentially improving the chances of a successful pregnancy [6].

This comprehensive review aims to delve into the pivotal role of transvaginal sonography in diagnosing female infertility. Transvaginal sonography, a non-invasive and widely accessible imaging technique, has become a cornerstone in evaluating female reproductive health. This review aims to thoroughly understand how transvaginal sonography is used to diagnose various causes of female infertility, including ovulatory disorders, structural abnormalities, endometrial issues, and more. Additionally, we will explore its advantages over other diagnostic methods, its limitations, and its role in improving patient outcomes.

## Review

Basics of transvaginal sonography

Transvaginal sonography, referred to as transvaginal ultrasound, is a medical imaging technique to visualize and assess the female reproductive organs and pelvic structures. It involves using a specialized ultrasound transducer, a transvaginal probe inserted into the vagina. Unlike traditional abdominal ultrasound, which involves placing the transducer on the surface of the abdomen, transvaginal sonography provides a closer and clearer view of the pelvic organs [7]. The transvaginal probe emits high-frequency sound waves that penetrate the vaginal walls and bounce off the internal structures, creating real-time images on a monitor. These images enable healthcare providers to examine the uterus, ovaries, fallopian tubes, cervix, and other nearby structures in detail. Transvaginal sonography offers superior image quality and greater sensitivity, making it a valuable tool for diagnosing various gynecological and reproductive conditions [7].

Advantages Over Other Diagnostic Methods

High-resolution imaging: One of its primary strengths is providing high-resolution, detailed images of the pelvic organs. This capability allows for detecting subtle abnormalities that might elude other imaging modalities. Whether identifying small ovarian cysts or characterizing uterine anomalies, the clarity of transvaginal sonography contributes significantly to diagnostic accuracy [8].

Non-invasive: Unlike invasive procedures like laparoscopy or hysterosalpingography (HSG), transvaginal sonography is non-invasive. Patients are spared the need for anesthesia or surgical intervention, reducing risks and minimizing discomfort. This non-invasiveness makes it appealing as an initial diagnostic option [9].

Real-time imaging: The real-time imaging capability of transvaginal sonography is a distinct advantage. It enables dynamic assessment, allowing clinicians to monitor processes such as follicular development, ovulation, and blood flow within the reproductive organs as they happen. This real-time insight aids in timing fertility treatments accurately and adapting interventions as needed [7].

Safety: Transvaginal sonography is generally safe and well-tolerated by most patients. Unlike modalities involving ionizing radiation, it relies on harmless ultrasound waves, making it suitable for repeated use, especially for monitoring the progress of fertility treatments over time. This safety profile minimizes patient concerns and enhances clinical utility [7].

Accessibility: Another critical advantage is the widespread availability of transvaginal sonography in healthcare facilities. This accessibility ensures patients have convenient access to this diagnostic tool, regardless of geographical location. It promotes timely evaluation and contributes to better patient outcomes [7].

Safety and Patient Comfort

Minimal discomfort: A transvaginal probe typically induces only mild discomfort or pressure. Patients often find it well-tolerated, and measures such as the use of lubrication and a gentle approach by the sonographer are employed to enhance comfort. The transient discomfort is outweighed by the valuable diagnostic insights it offers [10].

No radiation exposure: A critical safety advantage is the absence of ionizing radiation exposure during transvaginal sonography. Unlike some diagnostic imaging methods, such as X-rays or CT scans, which carry inherent radiation-related risks, this modality relies on harmless ultrasound waves. This feature is especially significant when repeated examinations are necessary, as in the monitoring of fertility treatments [11].

Non-invasive and no anesthesia: One of the most reassuring aspects for patients is that transvaginal sonography is entirely non-invasive. Patients are not subjected to anesthesia, surgical incisions, or invasive procedures. This minimizes more invasive diagnostic techniques' physical and psychological burdens [12].

Privacy and dignity: Ensuring patient comfort extends beyond the physical aspects. Healthcare providers strongly emphasize respecting patient privacy and dignity throughout the procedure. The examination is conducted discreetly and professionally, allowing patients to maintain their privacy and self-respect [13].

Causes of female infertility

Ovulatory Disorders

Polycystic ovary syndrome: Polycystic ovary syndrome (PCOS) is one of the most prevalent causes of female infertility. It is characterized by hormonal imbalances that disrupt the menstrual cycle and ovulation. Transvaginal sonography plays a pivotal role in diagnosing PCOS by revealing hallmark features within the ovaries. During the examination, multiple small follicular cysts, often likened to a "string of pearls," can be identified within the ovaries. These cysts result from arrested follicular development, which prevents the release of mature eggs. By visualizing these characteristic cysts, transvaginal sonography aids in confirming the diagnosis of PCOS, guiding treatment strategies, and assessing the response to interventions [14].

Hypothalamic-pituitary-ovarian axis dysfunction: Dysfunction within the hypothalamic-pituitary-ovarian (HPO) axis can be evaluated through transvaginal ultrasonography, a non-invasive imaging technique enabling the assessment of ovarian function and follicular development [15]. This dysfunction has implications for female fertility and ovulation, underscoring the importance of monitoring follicular development and detecting ovulation in evaluating ovarian reserve function [15]. Key parameters such as ovarian volume, antral follicle count, peak flow rate of the ovarian interstitial artery, and peak end-diastolic flow velocity are pivotal indicators [15]. Analyzing the correlation between follicle-stimulating hormone (FSH) levels, the FSH/luteinizing hormone (LH) ratio, and ultrasound indexes enhances our understanding of HPO axis function [15]. HPO axis dysfunction may manifest in symptoms like irregular ovulation, cystic ovaries, and other reproductive disorders [15]. Transvaginal ultrasonography is a valuable tool for assessing HPO axis dysfunction, providing comprehensive insights into ovarian function, follicular development, and ovulation. This information aids healthcare providers in identifying the underlying causes of fertility issues associated with HPO axis dysfunction, facilitating the development of targeted and effective treatment plans [15].

Structural Abnormalities

Uterine anomalies: Uterine anomalies encompass a diverse range of congenital abnormalities in the structure of the uterus. These anomalies can include conditions such as a septate uterus (a uterine septum dividing the uterine cavity), a bicornuate uterus (a uterus with two separate horns), or other variations in uterine shape. Uterine anomalies can potentially interfere with implantation or increase the risk of recurrent miscarriages. Transvaginal sonography offers a powerful diagnostic tool for assessing uterine abnormalities. By providing detailed and real-time visualization of the uterine cavity, this imaging technique allows healthcare providers to identify structural anomalies accurately. Such information is crucial for guiding treatment decisions, mainly when surgical correction is necessary to optimize fertility outcomes [16].

Fallopian tube disorders: Proper functioning of the fallopian tubes is essential for fertility, as these slender tubes are responsible for transporting eggs from the ovaries to the uterus and facilitating the meeting of sperm and egg for fertilization. Disorders affecting the fallopian tubes, such as blockages, scarring, or damage, can hinder the fertilization process and the passage of the fertilized embryo into the uterus. When used with other diagnostic techniques like HSG, transvaginal sonography is a valuable tool for assessing tubal patency and detecting abnormalities within the fallopian tubes. While transvaginal sonography may not directly visualize the fallopian tubes, it can provide insights into other pelvic structures that may indirectly suggest tubal issues. HSG or laparoscopy may be employed to comprehensively evaluate the fallopian tubes [17].

Endometrial Issues

Endometriosis: Endometriosis is a challenging condition in which tissue similar to the uterine lining (endometrium) grows outside the uterus, often in the pelvic cavity. This abnormal tissue growth can lead to pelvic pain and is a known cause of infertility. Transvaginal sonography plays a crucial role in diagnosing endometriosis by enabling the visualization of endometrial implants, cysts (endometriomas), and adhesions within the pelvis. During the examination, healthcare providers can identify these characteristic features, helping to confirm the diagnosis of endometriosis. Early detection through transvaginal sonography is essential for timely intervention and management of the condition, which can improve fertility outcomes for affected individuals [18].

Asherman's syndrome: Asherman's syndrome results from forming intrauterine adhesions or scar tissue within the uterine cavity. These adhesions typically develop following uterine surgeries, such as dilation and curettage, or as a result of uterine infections. Asherman's syndrome can obstruct the passage of sperm and interfere with embryo implantation. Transvaginal sonography is a valuable tool for assessing the uterine cavity and identifying the presence of adhesions. By visualizing these adhesions, transvaginal sonography contributes to diagnosing Asherman's syndrome. This information guides healthcare providers in developing appropriate treatment strategies, which may include surgical intervention to remove the adhesions and restore uterine health, ultimately improving fertility prospects for affected individuals [19].

Age-Related Infertility

Age-related infertility is a significant concern for women as they grow older. Advancing age can profoundly affect female fertility, primarily due to the natural aging process impacting the ovaries. Transvaginal sonography is valuable for assessing and understanding age-related fertility changes [20]. One of the primary factors contributing to age-related infertility is the decline in the quantity and quality of a woman's eggs. The ovaries contain a finite number of eggs; as a woman ages, this ovarian reserve diminishes. Transvaginal sonography plays a crucial role in evaluating ovarian reserve through several vital parameters:

Antral follicle count: Transvaginal sonography allows healthcare providers to conduct an antral follicle count. Antral follicles are small, immature ovarian follicles that can be visualized on ultrasound. The number of antral follicles detected during transvaginal sonography provides valuable information about a woman's ovarian reserve. A lower antral follicle count may indicate a reduced pool of available eggs, which can impact fertility potential [21].

Ovarian size and morphology: Transvaginal sonography also enables the assessment of ovarian size and morphology. Changes in ovarian size, such as a decrease in volume, can indicate age-related changes in ovarian function. Additionally, alterations in ovarian morphology, such as cysts or structural irregularities, can be identified through transvaginal sonography [22].

Other Contributing Factors

Hormonal imbalances: Hormonal imbalances, such as thyroid disorders (hypothyroidism or hyperthyroidism) or hyperprolactinemia (elevated levels of the hormone prolactin), can disrupt the delicate hormonal regulation of the menstrual cycle and ovulation. These imbalances can lead to irregular or absent ovulation, making conception difficult. Transvaginal sonography can be instrumental in detecting structural abnormalities within the pituitary gland or thyroid gland that may contribute to these hormonal imbalances. While it does not directly diagnose hormonal issues, transvaginal sonography provides valuable insights into these glands' health, aiding in identifying potential underlying causes of fertility challenges. This information guides healthcare providers in formulating appropriate treatment strategies to address hormonal imbalances and improve fertility prospects [23].

Lifestyle and environmental factors: Lifestyle factors, such as excessive stress, poor nutrition, smoking, excessive alcohol consumption, and exposure to environmental toxins, can exert a significant impact on female fertility. These factors can disrupt hormonal balance, affect the menstrual cycle, and contribute to fertility challenges. While transvaginal sonography does not directly diagnose lifestyle and environmental factors, it plays a role in ruling out structural and physiological causes of infertility. By eliminating structural issues as the primary cause, healthcare providers can guide patients toward a more comprehensive assessment of their overall health and well-being. This comprehensive approach may involve lifestyle modifications, nutritional guidance, stress management strategies, and environmental toxin avoidance to optimize fertility [24].

Transvaginal sonography technique

Preparation of the Patient

Informed consent: Begin by obtaining informed consent from the patient. During this process, thoroughly explain the nature and purpose of the transvaginal sonography procedure, ensuring the patient is fully aware of what to expect. Describe any potential discomfort, risks, or complications associated with the examination. Encourage the patient to ask questions and address any concerns before proceeding. Obtaining informed consent ensures ethical practice and fosters trust and cooperation between the patient and the healthcare provider [25].

Empty bladder: Instruct the patient to empty her bladder before the examination. A partially filled bladder can interfere with the sonographer's ability to obtain clear and accurate images of the pelvic organs. Furthermore, an empty bladder enhances patient comfort during the procedure, as it minimizes pressure on the bladder and reduces the likelihood of discomfort. Ensure the patient understands the importance of this step and provide ample time for her to use the restroom before the examination [26].

Positioning: Assist the patient in assuming the lithotomy position, a standard and widely used position for transvaginal sonography. In this position, the patient lies on her back with her feet in stirrups. The lithotomy position facilitates easy access for the transvaginal transducer, allowing the sonographer to obtain optimal imaging of the pelvic structures. Ensure the patient is comfortable and properly positioned, and provide any necessary support to maintain this posture throughout the examination [7].

Privacy and comfort: Prioritize the patient's privacy, dignity, and comfort throughout the procedure. Create a welcoming and confidential environment that fosters trust and reduces anxiety. Offer the patient a gown or drape to cover herself and maintain her modesty. Address any questions or concerns the patient may have, and provide clear instructions regarding the procedure's progression. Always maintain open communication with the patient, encouraging her to communicate discomfort or distress during the examination. Adhering to the principles of patient-centered care ensures a positive experience and practical cooperation during transvaginal sonography [27].

Equipment and Transducer Selection

Transvaginal transducer: Select a transvaginal probe or transducer designed explicitly for intravaginal use. These transducers are typically high-frequency, offering superior image resolution for gynecological examinations. Ensure that the transducer is in optimal working condition and has a sterile cover or has been thoroughly cleaned and disinfected according to established protocols before each examination. Patient safety and hygiene are paramount, and using a sterile cover or proper disinfection procedures minimizes the risk of infection [12].

Ultrasound machine: Ensure that the ultrasound machine used for transvaginal sonography works appropriately. Regular maintenance and calibration of the machine are essential to guarantee accurate and reliable imaging. The machine should also be set to the appropriate settings for gynecological examinations. This includes selecting the correct mode (such as B-mode or Doppler) and adjusting parameters like gain and frequency to optimize image quality [7].

Gel and lubrication: Have an appropriate ultrasound gel or lubricant readily available for the procedure. The gel is essential for facilitating the movement of the transducer and ensuring good acoustic contact with the patient's body. It also enhances patient comfort during the examination. Ensure the gel is suitable for transvaginal use and does not cause irritation [28].

Sterile covers and infection control: If using disposable sterile covers for the transvaginal transducer, ensure an adequate supply. If reusable transducers are employed, stringent infection control protocols must be followed to clean and disinfect the transducer between patients. Compliance with infection control measures is critical to prevent the transmission of infections and ensure patient safety [29].

Scanning Protocol and Procedures

Initial survey: The examination begins with an initial survey of the pelvis. During this phase, the sonographer systematically assesses the adnexa (which includes the ovaries and fallopian tubes), the uterus, and the surrounding pelvic structures. This comprehensive survey sets the foundation for further evaluation [30].

Ovarian assessment: Following the initial survey, the focus shifts to the ovaries. The sonographer evaluates the ovaries for size, shape, and the presence of any abnormalities. This assessment includes measuring the ovaries' size and inspecting for cysts, follicles, or other anomalies. In cases where ovarian reserve assessment is necessary, the antral follicle count is measured, providing valuable information about the quantity of available eggs [31].

Uterine assessment: The examination then proceeds to assess the uterus. The sonographer examines the uterus's size, shape, and position, looking for any deviations from normal. Additionally, the endometrial thickness is measured, which can provide insights into the menstrual cycle phase and potential causes of abnormal bleeding. Abnormalities within the uterine cavity, such as polyps or fibroids, are also carefully evaluated [32].

Fallopian tube assessment: If required, the tubes are assessed for patency and any structural abnormalities that might impede their function. Techniques like color Doppler may detect blood flow within the tubes. Assessing fallopian tube health is particularly important in cases where tubal factors may contribute to infertility [33].

Documentation: The sonographer meticulously documents all relevant measurements and findings throughout the examination. This documentation includes capturing images and, in some cases, videos that provide a visual record of the examination. These records are valuable references for diagnosis, treatment planning, and follow-up consultations [34].

Patient interaction: Effective communication with the patient is a continuous aspect of the procedure. The sonographer interacts with the patient, explaining each step of the examination, sharing findings in real-time, and addressing any concerns or discomfort the patient may experience. Open communication ensures the patient's comfort and informed participation and enhances the patient experience [35].

Interpretation of Sonographic Finding

Normal vs. abnormal findings: The first task in interpretation is to distinguish between normal and abnormal findings. This determination relies on established criteria and guidelines, considering factors such as the patient's age and menstrual cycle phase when relevant. Typical anatomical structures and physiological variations are assessed to provide context for identifying abnormalities [36].

Pathological conditions: When abnormal findings are identified, the sonographer carefully identifies and documents any pathological conditions present. These conditions may include ovarian cysts, uterine fibroids, endometrial polyps, anatomical anomalies (such as uterine septums or bicornuate uteri), or other abnormalities within the pelvic organs. Its location, size, and shape characterize each finding and other relevant characteristics to describe the pathology accurately [37].

Differential diagnosis: When abnormalities are identified during the sonographic examination, the sonographer typically focuses on reporting the sonographic findings. Subsequent consideration of potential differential diagnoses is usually within the purview of the clinician. This crucial step involves the clinician evaluating a range of clinical scenarios and conditions that could explain the observed sonographic abnormalities [38]. The differential diagnosis process plays a pivotal role in guiding the next steps of the diagnostic journey. Clinicians may decide on further investigations, such as additional imaging modalities like MRI or CT scans, or specific laboratory tests like hormonal assays [38]. By emphasizing the collaboration between sonographers and clinicians, this approach ensures a comprehensive assessment and aids in the formulation of an accurate diagnosis and tailored treatment plan.

Reporting: The sonographer compiles the interpretation of findings into a clear and comprehensive report. This report summarizes the findings, including measurements, descriptions of abnormalities, and any clinical implications. The report is a critical communication tool between the sonographer and the referring healthcare provider, ensuring that the provider receives all necessary information for clinical decision-making. The report is typically prepared promptly to facilitate timely patient care [39].

Follow-up and management: In collaboration with the patient's healthcare team, the sonographer determines appropriate follow-up and management plans based on the sonographic findings. This may involve consultations with gynecologists, reproductive endocrinologists, or other specialists. The information provided through transvaginal sonography contributes to developing individualized treatment strategies and interventions to address the underlying causes of female infertility. Follow-up examinations and assessments are scheduled as needed to monitor progress and evaluate treatment outcomes [40].

Role of transvaginal sonography in diagnosing female infertility

Ovulatory Disorders

Follicular monitoring, transvaginal sonography, is an invaluable tool for assessing follicular development, a central component of the ovulatory process. This monitoring process involves meticulously tracking ovarian follicles' growth and maturation throughout the menstrual cycle, typically commencing in the early follicular phase. The advantages of transvaginal sonography for follicular monitoring are well-documented in the literature [41]. One crucial aspect is its capacity to identify abnormal follicular development. Through high-resolution imaging, transvaginal sonography can pinpoint irregularities in follicular growth, such as the absence of dominant follicles or irregular growth patterns. This information is pivotal in diagnosing ovulatory disorders like anovulation or oligo-ovulation [42].

Additionally, transvaginal sonography plays a pivotal role in accurately timing ovulation. By closely monitoring follicular development, clinicians can predict and precisely time the occurrence of ovulation. This timing is of paramount importance for planning fertility treatments, including intrauterine insemination or in vitro fertilization (IVF), to maximize the chances of conception [5]. The technique also extends its utility to measuring the antral follicle count, representing the number of small, resting follicles in the ovaries. Antral follicle count offers valuable insights into ovarian reserve, indicating the quantity and quality of eggs remaining in the ovaries. A lower antral follicle count may suggest diminished ovarian reserve, a significant concern in infertility evaluation [21].

Furthermore, transvaginal sonography excels in detecting and evaluating ovarian cysts, whether functional or pathological (e.g., endometriomas). This capability yields several advantages, including precise identification and characterization of ovarian cysts. The high-resolution imaging provided by transvaginal sonography allows for accurately determining the cyst's presence, size, and exact location within the ovaries, essential for differentiating between benign and potentially malignant or complex cysts [43].

Moreover, transvaginal sonography enables longitudinal monitoring of ovarian cysts, a critical aspect of clinical practice. Ovarian cysts can undergo dynamic changes over time, and this technique facilitates the systematic tracking of these alterations. Clinicians can use transvaginal sonography to monitor shifts in cyst size, composition, and appearance throughout the menstrual cycle or over extended periods, aiding in distinguishing between simple, functional cysts and more complex or persistent cysts that may require medical or surgical management [44].

Structural abnormalities

Uterine Anomalies Assessment

Transvaginal sonography extends its diagnostic capabilities to the realm of reproductive health by detecting various uterine anomalies and conditions that can significantly impact fertility. First and foremost, it excels in identifying congenital uterine anomalies, such as septate or bicornuate uteri. These anomalies can disrupt fertility by altering the uterine structure, potentially leading to implantation difficulties or recurrent miscarriages. Transvaginal sonography's ability to visualize the internal uterine architecture in detail allows for the early diagnosis of these conditions, facilitating timely interventions and management [45].

Furthermore, the technique assesses uterine size, shape, and position within the pelvis. Deviations from normal uterine dimensions and orientation can indicate structural abnormalities that may impact fertility. For instance, a retroverted uterus can pose challenges during conception. Transvaginal sonography's capacity to capture these parameters comprehensively evaluates the uterine environment [46].

In addition to congenital anomalies and structural assessments, transvaginal sonography is crucial in identifying intrauterine adhesions, known as Asherman's syndrome. This condition arises due to the formation of scar tissue within the uterine cavity, potentially obstructing the passage of sperm or hindering embryo implantation. Moreover, the technique is adept at detecting uterine fibroids (leiomyomas) and benign growths within the uterine wall. Depending on their location and size, fibroids can interfere with fertility and may necessitate management or removal [47].

Tubal Patency Evaluation

Transvaginal sonography plays a critical role in assessing the condition of the fallopian tubes, which holds significant relevance in the context of fertility evaluation and management. One of its primary functions is the evaluation of fallopian tube patency. Through transvaginal sonography, clinicians can detect the presence of fluid accumulation within the fallopian tubes, a condition known as hydrosalpinx. Hydrosalpinx can disrupt the transport of both egg and sperm, thus impeding the chances of conception. Identifying hydrosalpinx using sonography provides crucial insights for clinicians when investigating fertility challenges [48].

Moreover, transvaginal sonography is adept at identifying tubal obstructions or abnormalities that may impede fertility. Such obstructions can result from scarring, infections, or previous surgeries. The technique's capacity to visualize the fallopian tubes and discern these issues aids in pinpointing potential causes of infertility [49].

Furthermore, transvaginal sonography is a pivotal guide in determining the following steps when concerns about tubal patency arise. In cases where abnormalities are detected, clinicians may recommend additional procedures such as HSG and contrast imaging studies to provide a more detailed assessment of tubal function. The insights gleaned from transvaginal sonography serve as a foundation for clinical decisions, ensuring that patients receive appropriate and timely interventions to address tubal-related fertility issues [50].

Endometrial Evaluation

Transvaginal sonography is a valuable diagnostic tool in detecting and assessing endometrial polyps, providing critical information for healthcare providers and patients. First, it offers high-resolution, real-time imaging that enables the clear visualization of endometrial polyps within the uterine cavity. These polyps appear as distinct, abnormal structures during the examination, allowing for their identification and characterization [51].

Beyond mere visualization, transvaginal sonography allows for precise measurements of the size and location of endometrial polyps. This quantitative data is essential for determining the severity of the polyp, its potential impact on fertility, and whether intervention, such as removal through polypectomy, is warranted to enhance fertility prospects. The ability to measure these parameters aids in assessing the urgency and necessity of clinical intervention [52].

Moreover, the visual and quantitative data obtained through transvaginal sonography serve as a cornerstone in guiding clinical decision-making. Healthcare providers can use this information to make informed decisions regarding managing endometrial polyps. Clinicians can determine the most appropriate course of action to optimize fertility outcomes depending on the polyp's size, location, and the patient's clinical context. This tailored approach ensures that patients receive individualized care that addresses their needs and concerns [53].

Assessment of Endometrial Thickness

Transvaginal sonography plays a crucial role in the assessment and monitoring of endometrial thickness throughout the menstrual cycle, particularly in the context of fertility treatments. First, it allows for measuring endometrial thickness during different menstrual cycle phases. This assessment is vital to ensure the endometrium achieves an optimal thickness conducive to successful embryo implantation. A thin or inadequate endometrial lining can present significant challenges to successfully establishing pregnancy [54].

Furthermore, in fertility treatments such as IVF, the ability to monitor changes in endometrial thickness is paramount. Transvaginal sonography facilitates real-time tracking of endometrial changes throughout the treatment cycle. Healthcare providers can closely monitor the thickness and receptivity of the endometrial lining, allowing them to adjust the timing of embryo transfer with precision. This personalized approach enhances the chances of successful implantation and subsequent pregnancy, ensuring the embryo is introduced into an environment optimal for implantation [55].

Monitoring Ovarian Reserve

Counting antral follicles: Transvaginal sonography allows the precise counting of antral follicles within the ovaries. Antral follicles are small, immature ovarian follicles that can be visualized on ultrasound. Healthcare providers can estimate the remaining eggs in a woman's ovaries by counting these antral follicles. A lower antral follicle count may indicate a reduced ovarian reserve, which can impact fertility potential. This information is instrumental in guiding treatment decisions, such as selecting fertility treatments or determining the optimal timing for assisted reproductive technologies (ART) [56].

Evaluating ovarian size and morphology: Transvaginal sonography enables the evaluation of ovarian size and morphology. Changes in ovarian size, such as a decrease in volume, can indicate age-related changes or conditions that may affect ovarian function. Additionally, alterations in ovarian morphology, such as cysts or structural irregularities, can be identified through transvaginal sonography. This comprehensive assessment provides insights into overall ovarian health, aiding healthcare providers in understanding potential factors affecting fertility [57].

Predicting response to ART: Transvaginal sonography assists clinicians in predicting a patient's response to ovarian stimulation protocols in ART, such as IVF. Transvaginal sonography helps tailor ART protocols to the individual patient by assessing the ovarian reserve and follicular development. Predicting the ovarian response ensures that the appropriate dosages of medications are administered, optimizing the chances of a successful IVF cycle and minimizing the risk of complications [58].

Identification of Other Contributing Factors

One of its vital roles is in identifying pelvic inflammatory disease (PID), an inflammatory condition of the female reproductive organs often caused by sexually transmitted infections or other infections. PID can lead to tubal scarring, pelvic pain, and infertility. Transvaginal sonography plays a crucial role in identifying signs of PID by enabling healthcare providers to visualize inflammatory changes in the pelvis. This includes the detection of fluid-filled pouches known as tubo-ovarian abscesses, which indicate severe pelvic inflammation and the potential for tubal damage. Assessing the extent and severity of pelvic inflammation through sonography provides valuable information for diagnosis and management, facilitating early intervention and treatment to prevent further damage to the reproductive organs [59].

Additionally, transvaginal sonography evaluates uterine fibroids (leiomyomas) and pelvic adhesions (scar tissue), which can negatively impact fertility outcomes. It allows healthcare providers to identify the presence, size, and location of uterine fibroids, which can distort the uterine cavity or block the fallopian tubes, potentially affecting fertility-accurate characterization of fibroids through sonography guides treatment decisions, including whether surgical intervention is necessary. Furthermore, in cases where fibroids are present, transvaginal sonography assesses their impact on the uterine cavity, as distortion of the uterine cavity can hinder embryo implantation and pregnancy. Sonographic findings aid in developing appropriate treatment strategies, such as myomectomy or hysteroscopic resection, to optimize fertility outcomes [60-62].

Moreover, transvaginal sonography is instrumental in detecting the presence of pelvic adhesions or scar tissue within the pelvic cavity, which can result from previous surgeries, infections, or conditions like endometriosis. Identifying pelvic adhesions through sonography guides healthcare providers in understanding potential sources of fertility challenges and aids in planning interventions to address them, helping individuals and couples on their journey to achieving a successful pregnancy [63-65].

Comparisons with other diagnostic methods

Hysterosalpingography

HSG and transvaginal sonography are two diagnostic methods used in gynecology to assess the uterine cavity and fallopian tubes. These techniques have distinct differences in imaging, uterine and tubal assessment, patient experience, and limitations [66]. Transvaginal sonography employs ultrasound waves to generate real-time images without exposing the patient to ionizing radiation. This non-invasive approach provides detailed visualization of the uterine cavity, allowing the detection of structural abnormalities, endometrial polyps, or fibroids. However, it has limitations in directly assessing tubal patency, as it can only indirectly detect fluid accumulation (hydrosalpinx) and may not offer definitive results [67].

In contrast, HSG involves using X-rays and a contrast dye, which exposes the patient to ionizing radiation. It primarily focuses on the uterine cavity but may not provide as detailed information about uterine anomalies as transvaginal sonography. However, it excels in directly evaluating tubal patency by visualizing the flow of contrast dye through the fallopian tubes, offering more conclusive results [68]. The patient experience differs between the two procedures as well. Transvaginal sonography is generally well-tolerated, with minimal discomfort associated with inserting the ultrasound probe and no exposure to radiation. In contrast, HSG may cause discomfort or cramping during the procedure, and some patients may experience allergic reactions to the contrast dye, making it a less comfortable option for some individuals [48].

Both techniques have their limitations. Transvaginal sonography is limited in directly assessing tubal patency and may miss subtle abnormalities. On the other hand, HSG is limited in providing detailed information about ovarian structures and exposes the patient to ionizing radiation, which can be a concern, particularly for women of reproductive age [69].

Laparoscopy

Laparoscopy and transvaginal sonography are contrasting approaches to assessing pelvic and abdominal organs, each offering unique advantages and disadvantages in gynecology [7]. Transvaginal sonography is a non-invasive technique that does not necessitate anesthesia or surgical incisions. It provides indirect visualization of pelvic structures through ultrasound, which can be valuable for identifying certain gynecological conditions but cannot directly inspect tissues. This procedure is generally associated with minimal post-procedure discomfort and allows for a rapid recovery. It is also more cost-effective and readily available compared to laparoscopy. However, its diagnostic range is limited to the pelvic region and may not effectively assess abdominal or retroperitoneal structures [70].

In contrast, laparoscopy is an invasive surgical procedure requiring anesthesia and small incisions to insert a laparoscope. This approach offers direct visualization and inspection of pelvic and abdominal organs. It is well-suited for detecting subtle abnormalities, adhesions, and conditions like endometriosis that may not be as easily identified through transvaginal sonography. Despite its diagnostic advantages, laparoscopy entails a more extended recovery period due to the surgical nature of the procedure. Additionally, it is more resource-intensive, requiring specialized surgical facilities, equipment, and skilled surgeons, which can make it a costlier option [71].

Blood Tests (Hormonal Assessments)

Blood tests and transvaginal sonography are valuable tools in assessing fertility, each offering distinct types of information and serving different purposes in the diagnostic process [72]. Using ultrasound to visualize pelvic structures, transvaginal sonography provides structural and anatomical information. It is beneficial in diagnosing structural abnormalities, ovarian pathologies, and endometrial conditions. However, it primarily focuses on the pelvic region and may not provide insights into systemic hormonal imbalances [70].

In contrast, blood tests are designed to measure hormonal levels related to fertility. They assess hormones such as FSH, LH, anti-Müllerian hormone, and estradiol, which offer critical insights into ovarian function and hormonal imbalances. Blood tests have a broader diagnostic range, as they can evaluate hormonal status across the entire endocrine system, providing valuable information about the patient's overall hormonal health [73]. Transvaginal sonography and blood tests are often used complementarily to assess female infertility. Transvaginal sonography can provide structural information about the uterus, ovaries, and fallopian tubes, while blood tests offer hormonal insights. Together, these diagnostic tools provide a more comprehensive evaluation of the patient's reproductive health [7].

Timing is an important consideration when comparing these methods. Transvaginal sonography provides real-time information about pelvic structures, allowing for dynamic assessment during the menstrual cycle. On the other hand, blood tests offer a snapshot of hormonal levels at the specific time the test is performed, often requiring multiple blood draws at different points in the menstrual cycle to gather comprehensive data [74]. In terms of cost and convenience, transvaginal sonography is typically less expensive and more readily available, and it can be performed at various stages of the menstrual cycle. Blood tests may involve multiple blood draws and require specific timing in the menstrual cycle, which can be less convenient for some patients [75].

Limitations and challenges of transvaginal sonography

Operator Dependence

Experience and skill: Transvaginal sonography requires high skill and experience for operators. It involves the skillful insertion and manipulation of an ultrasound probe within the patient's vagina to obtain clear images of pelvic structures. Inexperienced or untrained operators may need help to achieve the necessary precision, resulting in suboptimal image quality and potentially inaccurate interpretations of findings. Skilled operators are better equipped to recognize normal anatomy and identify abnormalities [7].

Subjectivity: The interpretation of sonographic findings can be subjective, and different operators may provide varying assessments of the same images. This subjectivity arises from differences in training, experience, and personal judgment. As a result, there is potential variability in the diagnosis or evaluation of conditions, which can affect patient care. Standardized protocols and guidelines are often used to mitigate this subjectivity to assist operators in their assessments [76].

Technical proficiency: Properly adjusting the ultrasound settings, selecting the appropriate transducer (probe), and effectively maneuvering the transducer within the vagina are essential for obtaining clear and accurate images during transvaginal sonography. Technical proficiency is crucial in optimizing image quality and ensuring that critical anatomical structures are adequately visualized. Operators must be well-versed in their equipment and possess the skills to conduct the examination efficiently and effectively [7].

Patient Factors

Patient discomfort: Transvaginal sonography involves the insertion of an ultrasound probe into the vagina, which can be uncomfortable or even painful for some patients, especially those with conditions like vaginismus (involuntary muscle spasms of the vaginal wall) or pelvic pain. This discomfort can lead to patient anxiety, reduced cooperation, and suboptimal image acquisition. Healthcare providers should be attentive to patient comfort and provide support to minimize discomfort during the procedure.

Obesity: In obese patients, excess abdominal and pelvic adipose tissue can present challenges for transvaginal sonography. The increased tissue thickness can impede the penetration of ultrasound waves and hinder the visualization of pelvic structures. Operators may need to adjust techniques, use specialized transducers, or consider alternative imaging modalities to overcome these challenges when evaluating obese patients.

Anatomical variations: Variability in pelvic anatomy among individuals is common and can pose challenges during transvaginal sonography. Pelvic structures may have different positions, orientations, or sizes, making operators need to adapt their approach to obtain optimal images. Operators must be skilled in recognizing and accommodating these anatomical variations to ensure accurate assessments.

Limited Visualization of Certain Structures

Deep pelvic structures: Transvaginal sonography is limited in visualizing deep pelvic structures in the retroperitoneal space or behind the uterus. Conditions affecting these areas may not be adequately assessed using transvaginal sonography alone, necessitating alternative imaging modalities or laparoscopy for a more comprehensive evaluation.

Intestinal gas: Intestinal gas in the abdomen can create acoustic artifacts and interfere with the visualization of adjacent structures. This limitation can make it challenging to accurately assess areas near the bowel, and it may require the patient to prepare by fasting or altering their diet to reduce gas before the examination.

Small lesions: Small and subtle lesions or abnormalities may not readily appear on transvaginal sonography. These can include tiny ovarian cysts, early-stage tumors, or small uterine fibroids. Additional imaging modalities, such as MRI, CT scans, or even laparoscopy, may be needed for accurate detection and characterization.

Scar tissue: Scar tissue resulting from previous surgeries or PID can obscure the visualization of nearby structures. This can pose diagnostic challenges, particularly when assessing the integrity of the uterus, fallopian tubes, or ovaries. Careful interpretation and consideration of clinical history are essential in these situations.

Tubal assessment: Transvaginal sonography can provide indirect clues to tubal patency by detecting fluid accumulation (hydrosalpinx), but it may not reliably confirm or rule out tubal obstruction. For a definitive assessment of the fallopian tubes, procedures like HSG or laparoscopy may be required.

Endometrial assessment: Transvaginal sonography detects common endometrial abnormalities such as polyps. However, subtle changes or small lesions within the endometrial lining may be challenging to visualize. In such cases, a hysteroscopy procedure that involves direct visualization of the uterine cavity with a thin, lighted tube may be necessary for a more detailed assessment.

## Conclusions

In conclusion, transvaginal sonography stands as a cornerstone in the diagnostic arsenal for female infertility, offering a non-invasive and real-time window into the complex world of pelvic health. Its significance lies in its versatility, enabling the detection of ovulatory disorders, structural anomalies, endometrial conditions, and more. As we look toward the future, the continual evolution of sonographic technology promises even greater precision and diagnostic capability. However, the true power of transvaginal sonography is realized when coupled with a proactive approach to early diagnosis and timely intervention. By encouraging individuals and couples to seek medical guidance at the onset of fertility concerns, we pave the way for tailored treatment plans, fertility preservation, and, ultimately, the fulfillment of the cherished dream of parenthood.

## References

[1] Greil AL, McQuillan J (2010). "Trying" times: medicalization, intent, and ambiguity in the definition of infertility. Med Anthropol Q.

[2] Sarkar S, Gupta P (2016). Socio-demographic correlates of women’s infertility and treatment seeking behavior in india. J Reprod Infertil.

[3] (2023). Infertility. https://www.who.int/news-room/fact-sheets/detail/infertility.

[4] (2023). 1 in 6 people globally affected by infertility: WHO. https://www.who.int/news/item/04-04-2023-1-in-6-people-globally-affected-by-infertility.

[5] Carson SA, Kallen AN (2021). Diagnosis and management of infertility: a review. JAMA.

[6] (2014). Female age-related fertility decline. Committee opinion no. 589. Fertil Steril.

[7] Nahlawi S, Gari N (2023). Sonography Transvaginal Assessment, Protocols, and Interpretation. StatPearls [Internet].

[8] Chandramohan A, Bhat TA, John R, Simon B (2020). Multimodality imaging review of complex pelvic lesions in female pelvis. Br J Radiol.

[9] Tan J, Deng M, Xia M, Lai M, Pan W, Li Y (2021). Comparison of hysterosalpingography with laparoscopy in the diagnosis of tubal factor of female infertility. Front Med (Lausanne).

[10] Merz E (2016). Is transducer hygiene sufficient when vaginal probes are used in the clinical routine?. Ultraschall Med.

[11] Lin EC (2010). Radiation risk from medical imaging. Mayo Clin Proc.

[12] Kaur A, Kaur A (2011). Transvaginal ultrasonography in first trimester of pregnancy and its comparison with transabdominal ultrasonography. J Pharm Bioallied Sci.

[13] Kadivar M, Mardani-Hamooleh M, Kouhnavard M (2018). Concept analysis of human dignity in patient care: Rodgers' evolutionary approach. J Med Ethics Hist Med.

[14] Zeng LH, Rana S, Hussain L (2022). Polycystic ovary syndrome: a disorder of reproductive age, its pathogenesis, and a discussion on the emerging role of herbal remedies. Front Pharmacol.

[15] Mikhael S, Punjala-Patel A, Gavrilova-Jordan L (2019). Hypothalamic-pituitary-ovarian axis disorders impacting female fertility. Biomedicines.

[16] Jayaprakasan K, Ojha K (2022). Diagnosis of congenital uterine abnormalities: practical considerations. J Clin Med.

[17] Han J, Sadiq NM (2023). Anatomy, Abdomen and Pelvis: Fallopian Tube. StatPearls [Internet].

[18] Tsamantioti ES, Mahdy H (2023). Endometriosis. StatPearls [Internet].

[19] Smikle C, Yarrarapu SNS, Khetarpal S (2023). Asherman Syndrome. StatPearls [Internet].

[20] Owen A, Sparzak PB (2023). Age-Related Fertility Decline. StatPearls [Internet].

[21] Agarwal A, Verma A, Agarwal S, Shukla RC, Jain M, Srivastava A (2014). Antral follicle count in normal (fertility-proven) and infertile Indian women. Indian J Radiol Imaging.

[22] Bachanek M, Abdalla N, Cendrowski K, Sawicki W (2015). Value of ultrasonography in the diagnosis of polycystic ovary syndrome - literature review. J Ultrason.

[23] Bahar A, Akha O, Kashi Z, Vesgari Z (2011). Hyperprolactinemia in association with subclinical hypothyroidism. Caspian J Intern Med.

[24] Agarwal A, Aponte-Mellado A, Premkumar BJ, Shaman A, Gupta S (2012). The effects of oxidative stress on female reproduction: a review. Reprod Biol Endocrinol.

[25] Baheti AD, Thakur MH, Jankharia B (2017). Informed consent in diagnostic radiology practice: where do we stand?. Indian J Radiol Imaging.

[26] Benacerraf BR, Shipp TD, Bromley B (2000). Is a full bladder still necessary for pelvic sonography?. J Ultrasound Med.

[27] (2023). Patient privacy, dignity and the importance of draping. College of physiotherapists of ontario. https://www.collegept.org/blog/post/college-blog/2019/04/11/patient-privacy-dignity-and-the-importance-of-draping.

[28] Afzal S, Zahid M, Rehan ZA (2022). Preparation and evaluation of polymer-based ultrasound gel and its application in ultrasonography. Gels.

[29] (2023). Healthcare equipment | disinfection & sterilization guidelines | guidelines library | infection control | CDC. https://www.cdc.gov/infectioncontrol/guidelines/disinfection/healthcare-equipment.html.

[30] Dewald O, Khan YS (2023). Sonography Gynecology Anatomy and Physiology. StatPearls [Internet].

[31] Kondagari L, Kahn J, Singh M (2023). Sonography Gynecology Infertility Assessment, Protocols, and Interpretation. StatPearls [Internet].

[32] Van den Bosch T (2012). Ultrasound in the diagnosis of endometrial and intracavitary pathology: an update. Australas J Ultrasound Med.

[33] Allahbadia GN (1993). Fallopian tube patency using color doppler. Int J Gynaecol Obstet.

[34] (2016). Proceedings of the British medical ultrasound society 47th annual scientific meeting 9-11 December 2015, City Hall, Cardiff, UK. Ultrasound.

[35] Kourkouta L, Papathanasiou IV (2014). Communication in nursing practice. Mater Sociomed.

[36] Committee on Diagnostic Error in Health Care; Board on Health Care Services; Institute of Medicine; The National Academies of Sciences, Engineering Engineering, and Medicine (2015). Improving Diagnosis in Health Care. https://www.ncbi.nlm.nih.gov/books/NBK338596/.

[37] Boyd CA, Riall TS (2012). Unexpected gynecologic findings during abdominal surgery. Curr Probl Surg.

[38] Cook CE, Décary S (2020). Higher order thinking about differential diagnosis. Braz J Phys Ther.

[39] Necas M (2018). The clinical ultrasound report: guideline for sonographers. Australas J Ultrasound Med.

[40] Thomas S, O'Loughlin K, Clarke J (2020). Sonographers' communication in obstetrics: challenges to their professional role and practice in Australia. Australas J Ultrasound Med.

[41] Debnath J, Satija L, Rastogi V (2000). Transvaginal sonographic study of follicular dynamics in spontaneous and clomiphene citrate cycles. Med J Armed Forces India.

[42] Jarrett BY, Vanden Brink H, Oldfield AL, Lujan ME (2020). Ultrasound characterization of disordered antral follicle development in women with polycystic ovary syndrome. J Clin Endocrinol Metab.

[43] Theodoridis TD, Zepiridis L, Mikos T, Grimbizis GF, Dinas K, Athanasiadis A, Bontis JN (2009). Comparison of diagnostic accuracy of transvaginal ultrasound with laparoscopy in the management of patients with adnexal masses. Arch Gynecol Obstet.

[44] Jung SI (2015). Ultrasonography of ovarian masses using a pattern recognition approach. Ultrasonography.

[45] Saravelos SH, Cocksedge KA, Li TC (2008). Prevalence and diagnosis of congenital uterine anomalies in women with reproductive failure: a critical appraisal. Hum Reprod Update.

[46] Esmaelzadeh S, Rezaei N, HajiAhmadi M (2004). Normal uterine size in women of reproductive age in northern Islamic Republic of Iran. East Mediterr Health J.

[47] Dreisler E, Kjer JJ (2019). Asherman's syndrome: current perspectives on diagnosis and management. Int J Womens Health.

[48] Panchal S, Nagori C (2014). Imaging techniques for assessment of tubal status. J Hum Reprod Sci.

[49] Ambildhuke K, Pajai S, Chimegave A, Mundhada R, Kabra P (2022). A review of tubal factors affecting fertility and its management. Cureus.

[50] Xu Z, Wang Y, Sun J, Chen S, Yan Z, Lin C, Shu J (2023). Evaluation of tubal patency by hysterosalpingo-contrast sonography (HyCoSy): controversies, dilemmas and considerations. Heliyon.

[51] Nijkang NP, Anderson L, Markham R, Manconi F (2019). Endometrial polyps: pathogenesis, sequelae and treatment. SAGE Open Med.

[52] Zhu H, Fu J, Lei H, Song Y, Shen L, Huang W (2016). Evaluation of transvaginal sonography in detecting endometrial polyps and the pregnancy outcome following hysteroscopic polypectomy in infertile women. Exp Ther Med.

[53] Vitale SG, Haimovich S, Laganà AS, Alonso L, Di Spiezio Sardo A, Carugno J (2021). Endometrial polyps. An evidence-based diagnosis and management guide. Eur J Obstet Gynecol Reprod Biol.

[54] Zhang CH, Chen C, Wang JR, Wang Y, Wen SX, Cao YP, Qian WP (2022). An endometrial receptivity scoring system basing on the endometrial thickness, volume, echo, peristalsis, and blood flow evaluated by ultrasonography. Front Endocrinol (Lausanne).

[55] McWilliams GD, Frattarelli JL (2007). Changes in measured endometrial thickness predict in vitro fertilization success. Fertil Steril.

[56] Coelho Neto MA, Ludwin A, Borrell A (2018). Counting ovarian antral follicles by ultrasound: a practical guide. Ultrasound Obstet Gynecol.

[57] Vanden Brink H, Jarrett BY, Pereira N, Spandorfer SD, Hoeger KM, Lujan ME (2023). Diagnostic performance of ovarian morphology on ultrasonography across anovulatory conditions - impact of body mass index. Diagnostics (Basel).

[58] Lai Q, Chen C, Zhang Z (2013). The significance of antral follicle size prior to stimulation in predicting ovarian response in a multiple dose GnRH antagonist protocol. Int J Clin Exp Pathol.

[59] Jennings LK, Krywko DM (2023). Pelvic Inflammatory Disease. StatPearls [Internet].

[60] Kairys N, Roepke C (2023). Tubo-Ovarian Abscess. StatPearls [Internet].

[61] Taipale P, Tarjanne H, Ylostalo P (1995). Transvaginal sonography in suspected pelvic inflammatory disease. Ultrasound Obstet Gynecol.

[62] Freytag D, Günther V, Maass N, Alkatout I (2021). Uterine fibroids and infertility. Diagnostics (Basel).

[63] Huang D, Magaoay B, Rosen MP, Cedars MI (2023). Presence of fibroids on transvaginal ultrasonography in a community-based, diverse cohort of 996 reproductive-age female participants. JAMA Netw Open.

[64] Guo XC, Segars JH (2012). The impact and management of fibroids for fertility: an evidence-based approach. Obstet Gynecol Clin North Am.

[65] Guerriero S, Ajossa S, Lai MP, Mais V, Paoletti AM, Melis GB (1997). Transvaginal ultrasonography in the diagnosis of pelvic adhesions. Hum Reprod.

[66] Acholonu UC, Silberzweig J, Stein DE, Keltz M (2011). Hysterosalpingography versus sonohysterography for intrauterine abnormalities. JSLS.

[67] Niknejadi M, Haghighi H, Ahmadi F, Niknejad F, Chehrazi M, Vosough A, Moenian D (2012). Diagnostic accuracy of transvaginal sonography in the detection of uterine abnormalities in infertile women. Iran J Radiol.

[68] Bhoil R, Sood D, Sharma T (2016). Contrast intravasation during hysterosalpingography. Pol J Radiol.

[69] Mayer C, Deedwania P (2023). Hysterosalpingogram. Treasure Island (FL).

[70] Karena ZV, Mehta AD (2023). Sonography Female Pelvic Pathology Assessment, Protocols, and Interpretation. StatPearls [Internet].

[71] Kavic SM, Kavic SM (2002). Adhesions and adhesiolysis: the role of laparoscopy. JSLS.

[72] Hrehorcak M, Nargund G (2011). "One-Stop” fertility assessment using advanced ultrasound technology. Facts Views Vis Obgyn.

[73] Oduwole OO, Huhtaniemi IT, Misrahi M (2021). The roles of luteinizing hormone, follicle-stimulating hormone and testosterone in spermatogenesis and folliculogenesis revisited. Int J Mol Sci.

[74] Hajishaiha M, Ghasemi-Rad M, Karimpour N, Mladkova N, Boromand F (2011). Transvaginal sonographic evaluation at different menstrual cycle phases in diagnosis of uterine lesions. Int J Womens Health.

[75] Campbell S (2013). A short history of sonography in obstetrics and gynaecology. Facts Views Vis Obgyn.

[76] Maxim LD, Niebo R, Utell MJ (2014). Screening tests: a review with examples. Inhal Toxicol.

